# Traffic Flow Prediction Based on Hypergraph Spatiotemporal Interaction Network

**DOI:** 10.3390/e28060664

**Published:** 2026-06-10

**Authors:** Wei Cao, Haipeng Jiang, Xinye Wu

**Affiliations:** 1 School of Computer and Information Engineering, Xiamen University of Technology, Xiamen 361024, China; 2 College of Architecture and Civil Engineering, Xiamen University, Xiamen 361005, China

**Keywords:** traffic flow prediction, dynamic spatio-temporal modeling, self-attention mechanism, hypergraph neural network, adaptive feature fusion

## Abstract

To improve the accuracy and stability of short-term traffic flow prediction in complex road networks and address the limitations of existing models in modeling spatiotemporal dependencies, this paper proposes a traffic flow prediction model based on a Hypergraph Spatio-Temporal Interaction Network (HGSTIN) in the context of intelligent transportation systems. The study constructs a multi-dimensional traffic pattern input tensor by integrating three temporal scales—proximity, intra-day, and intra-week—while taking traffic flow as the prediction target and introducing average speed and lane occupancy as auxiliary features. In terms of temporal modeling, a Transformer architecture integrated with a Dynamic Tanh (DyT) mechanism is adopted to capture multi-period variations. For spatial modeling, a neighborhood hypergraph and a DTW-based semantic hypergraph are combined to enhance the representation of local and global through spatial self-attention and hypergraph neural network branches, and an adaptive feature fusion module is designed to perform adaptively weighted fusion of the outputs from the two branches. In terms of loss function design, a temporal gradient consistency loss function is proposed to enhance the robustness of predictions. Experimental results on the PEMS04 and PEMS08 datasets show that the proposed model achieves average improvements of approximately 5.15%, 1.76%, and 3.88% in MAE, RMSE, and MAPE, respectively, compared to the second-best baseline model. The model exhibits the smallest performance degradation in multi-step prediction scenarios, and the effectiveness of each module is validated through ablation studies. The findings demonstrate that HGSTIN can effectively capture the dynamic spatiotemporal characteristics of complex traffic scenarios, thereby providing high-precision prediction support for intelligent transportation systems.

## 1. Introduction

With the acceleration of urbanization and the improvement of living standards, traffic pressure in Chinese cities has been increasing daily. The growth of traffic infrastructure has struggled to keep pace with demand, leading to intensified congestion, reduced efficiency, increased energy consumption, and worsening environmental pollution. Traditional methods, such as expanding road networks, are difficult to implement effectively due to long construction cycles and high costs. Meanwhile, the increasing complexity of traffic systems and the unpredictability of incidents—such as accidents, roadwork, and adverse weather—have heightened management challenges, driving the continuous evolution of traffic prediction technologies. Intelligent Transportation Systems (ITS) have emerged as a solution, integrating sensing, communication, computing, and control technologies to obtain and regulate real-time traffic flow information, thereby significantly enhancing road efficiency and safety. Within ITS, traffic flow prediction is a core technology that analyzes historical and real-time data to forecast future traffic conditions, providing a critical basis for traffic management and route optimization. Traffic flow prediction involves estimating potential traffic variations over a short period to determine changes in vehicle volume [1]. High-quality forecasting not only helps alleviate congestion and optimize scheduling but also improves the travel experience and reduces energy consumption, carrying significant practical importance.

Early traffic flow prediction primarily relied on mathematical and statistical methods [2], which offer advantages such as simple modeling and high computational efficiency. However, these methods generally depend on the assumption of traffic flow stationarity, making it difficult to handle sudden incidents or complex non-linear relationships. In recent years, deep learning technologies—including Convolutional Neural Networks (CNNs), Recurrent Neural Networks (RNNs), and Multi-Layer Perceptrons (MLPs)—have demonstrated strong performance in extracting spatio-temporal features from traffic data. Graph Neural Networks (GNNs) have been widely applied to model road network topologies, showing effectiveness in establishing spatial correlations [3]. Graph Convolutional Networks (GCNs), an extension of GNNs, extract node features through convolutional operations. Nevertheless, the adjacency matrices used in traditional GCNs are typically predefined and cannot be dynamically adjusted based on real-time traffic conditions, limiting their flexibility in modeling road relationships. To address this, Yu et al. [4] proposed Spatio-Temporal Graph Convolutional Networks (STGCNs), which dynamically optimize predefined road correlations during the training phase. Considering global spatio-temporal correlations, Song et al. [5] introduced the STSGCN model, utilizing synchronous spatio-temporal graph convolutional modules to effectively extract localized heterogeneous features. Feng et al. [6] proposed Hypergraph Neural Networks (HGNNs) to characterize road networks using hypergraph structures. The HGNN effectively captures correlations between multimodal and complex data while maintaining low computational complexity, outperforming mainstream models like GCN in tasks such as citation network classification and 3D object recognition. Recently, Hu et al. [7] proposed PDR-STGCN for traffic forecasting. The model uses periodicity-aware embeddings to capture daily and weekly traffic patterns and employs a dynamic relational graph to learn time-varying spatial dependencies among road nodes, demonstrating strong performance on multiple traffic datasets. However, PDR-STGCN mainly focuses on dynamic pairwise graph relations and periodic feature embeddings, without explicitly modeling higher-order spatial dependencies among multiple traffic nodes.

In addition to spatial dependency modeling, capturing long-range temporal dependencies is also crucial for accurate traffic flow prediction. The Transformer, an architecture built upon the self-attention mechanism, is well-suited for processing data with long-term temporal dependencies. Research has proposed combining the Transformer with a multi-head attention mechanism for traffic flow prediction [8]. Comparative experiments with traditional RNN models, such as GRU and LSTM, indicate that this approach excels at capturing long-range dependencies in time series. Wen et al. [9] introduced relative position encoding and optimized the multi-head attention structure via linear mapping to more effectively mine the relative positional relationships between sequence nodes. Zhu et al. [10] proposed a novel Transformer structure that eliminates the need for normalization, termed Dynamic Tanh (DyT). In the non-linear mapping dimension, element-wise operations based on functions are designed to capture extreme value compression characteristics typically handled by normalization; in the parameterized scaling dimension, learnable scalars are introduced to dynamically adjust the input range. Although Transformer models perform well in traffic prediction, their accuracy can decline significantly when forecasting longer-term conditions, as they may struggle to fully capture the underlying spatio-temporal evolutionary patterns.

Existing research in traffic flow prediction reveals limitations in capturing dynamic spatio-temporal dependencies and handling long-sequence data. To address the dynamic spatial correlations and long-term cross-spatial dependencies inherent in traffic data, this study designs a Hypergraph Spatio-Temporal Interaction Network (HGSTIN) to effectively capture the temporal evolution and long-term dependencies of traffic flow.

This study does not claim novelty for each individual component. Instead, the main novelty of HGSTIN lies in the framework-level integration of dual hypergraph branches and adaptive fusion, which jointly model local physical adjacency and global temporal-pattern similarity for traffic flow prediction. The primary contributions are as follows:

(1) We propose a Hypergraph Spatio-Temporal Interaction Network (HGSTIN) for traffic flow prediction. Instead of claiming novelty for each individual component, this study emphasizes the integration of dual hypergraph branches and adaptive fusion within a unified spatio-temporal forecasting framework.
(2) We construct two complementary hypergraph branches to model different types of spatial dependencies. The neighborhood hypergraph captures local physical adjacency among traffic sensors, while the DTW-based semantic hypergraph captures global similarity in temporal traffic patterns. These two branches provide complementary views of spatial dependency modeling.
(3) We introduce an adaptive fusion mechanism to combine the representations learned from the neighborhood and semantic hypergraph branches. This allows the model to adjust the relative importance of local physical dependencies and global semantic dependencies under different traffic conditions.
(4) We incorporate a temporal gradient consistency loss to encourage the predicted traffic sequence to preserve the temporal variation tendency of the ground truth. Extensive experiments on PEMS04 and PEMS08 demonstrate the effectiveness of the proposed framework, and ablation studies further verify the contribution of the dual hypergraph branches, adaptive fusion module, and temporal consistency constraint.

## 2. Problem Description and Preliminaries

### 2.1. Topological Modeling of Traffic Networks

In this study, the traffic network is defined as a graph G=(V,E,A), where $V=\{v_{1},v_{2},\ldots ,v_{N}\}$ represents the set of N ertices (sensors) in the road network, and the set E denotes the edges connecting any two road nodes. $A\in R^{N\times N}$ is the adjacency matrix used to describe the connectivity between nodes in graph G. For any two nodes $v_{i},v_{j}\in V$, if an edge $(v_{i},v_{j})\in E$ exists (indicating a direct physical connection), the element $a_{ij}$ is set to 1; otherwise, it is 0. Since sensor data in actual road networks reflects the overall state of road segments, the adjacency matrix is defined as a symmetric matrix, thereby ignoring directionality and retaining only physical connectivity features.

Traffic flow data comprises three core features: traffic volume, average speed, and lane occupancy. Average speed and lane occupancy serve as auxiliary features to provide essential spatio-temporal information for the model. Lane occupancy helps the model identify congestion levels across different segments, particularly in high-density areas and traffic bottlenecks, reflecting the spatial dependencies between adjacent segments. Average speed provides information on road efficiency and assists the model in capturing the dynamic variations of traffic flow, especially under conditions of high volume or sudden incidents, thereby enhancing the model’s adaptability to complex scenarios.

Incorporating these auxiliary features allows the model to more accurately capture changes in spatial characteristics, improving prediction precision and robustness in complex traffic environments. In this paper, traffic volume is the primary prediction target, while speed and lane occupancy are utilized as auxiliary features.

At time step t, the traffic data forms a feature matrix $X_{\left(t\right)}=(X_{1t},X_{2t},\ldots ,X_{Nt}{)}^{T}\;\in R^{N\times F}$, where $X_{\left(t\right)}$ represents the values of all features for all nodes at time t, and F denotes the feature dimension. The primary input feature is traffic volume, expressed as follows:

(1) $$X_{\left(t\right)}=\left[F_{1}\;|\;F_{2}\;|\;F_{3}\right]\in R^{N\times \left(F_{1}+F_{2}+F_{3}\right)}$$

where $F=F_{1}+F_{2}+F_{3}$, $F_{1}$ is the primary feature (traffic volume), while $F_{2}$ (average speed) and $F_{3}$ (lane occupancy) are auxiliary features.

Given the spatial graph G and historical traffic data collected over $T_{r}$ consecutive time steps, the task of traffic flow prediction is to forecast the traffic state for the next $T_{k}$ time steps. This can be formalized as follows:

(2) $$f\left(\left[X_{\left(t-T_{r}+1\right):t},G\right]\right)\to X_{\left(t+1\right):\left(t+T_{k}\right)}$$

where $X_{\left(t-T_{r}+1\right):t}\in R^{B\times N\times F\times T_{r}}$ represents the data for $T_{r}$ historical time steps required by the model, $X_{\left(t+1\right):\left(t+T_{k}\right)}\in R^{B\times N\times F\times T_{k}}$ is the predicted output, B is the batch size, and f denotes the spatio-temporal fusion prediction model.

### 2.2. Processing of Data Periodicity

In the construction of traffic flow prediction models, the introduction of periodicity is of critical significance, as it substantially enhances the accuracy and reliability of prediction results [11].

In addition to using historical observations from the most recent $T_{r}$ time steps, this study incorporates daily and weekly periodic features to strengthen the model’s ability to capture temporal dependencies. The specific definitions are as follows:

(1) Proximity Data Source

**Figure 1:**
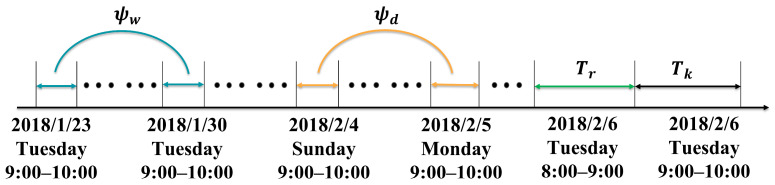
Example diagram of input periodic sequence.

The proximity data source ψ is introduced using the past $T_{r}$ time steps, as shown in the ψ section of Figure 1.

(3) $$\psi ={(X}_{t_{0}-T_{r}+1},\ldots ,X_{t_{0}})\in R^{B\times N\times F\times T_{r}}$$

where $t_{0}$ represents the time of the prediction slice.

(2) Daily Periodic Data Source

The $T_{k}$ segments from the traffic records of each of the past d consecutive days are introduced as the daily periodic data source $\psi_{d}$, as shown in the $\psi_{d}$ section of Figure 1.

(4) $$\psi_{d}=\left(X_{t_{0}-d*q+1},\ldots ,X_{t_{0}-d*q+T_{k}},X_{t_{0}-\left(d-1\right)*q+1},\ldots ,X_{t_{0}-\left(d-1\right)*q+T_{k}},\ldots ,X_{t_{0}-q+1},\ldots ,X_{t_{0}-d*q+T_{k}}\right)$$

where $\psi_{d}\in R^{B\times N\times F\times d*T_{k}}$, and q represents the number of time steps in a 24-h day.

(3) Weekly Periodic Data Source

The $T_{k}$ segments from the traffic records of the same day over the past w weeks are introduced as the weekly periodic data source $\psi_{w}$, as shown in the $\psi_{w}$ section of Figure 1.

(5) $$\begin{array}{c} \psi_{w}={(X}_{t_{0}-7*w*q+1},\ldots ,X_{t_{0}-7*w*q+T_{k}},X_{t_{0}-7*\left(w-1\right)*q+1},\ldots ,\\ X_{t_{0}-7*\left(w-1\right)*q+T_{k}},\ldots ,X_{t_{0}-7*q+1},\ldots ,X_{t_{0}-7*q+T_{k}}) \end{array}$$

where $\psi_{w}\in R^{B\times N\times F\times w*T_{k}}$.

After obtaining the proximity tensor ψ, daily periodic tensor $\psi_{d}$, and weekly periodic tensor $\psi_{w}$, they are concatenated to form the model input $\psi_{\mathrm{data}}=(\psi \left\|\psi_{d}\right\|\psi_{w})\in R^{B\times N\times F\times (T_{r}+d*T_{k}+w*T_{k})}$, as illustrated in Figure 1.

All features undergo unified data preprocessing before concatenation. The input traffic data includes volume, speed, and lane occupancy across three periodic scales, resulting in an input feature dimension of 9.

## 3. Hypergraph Spatio-Temporal Interaction Network (HGSTIN)

**Figure 2:**
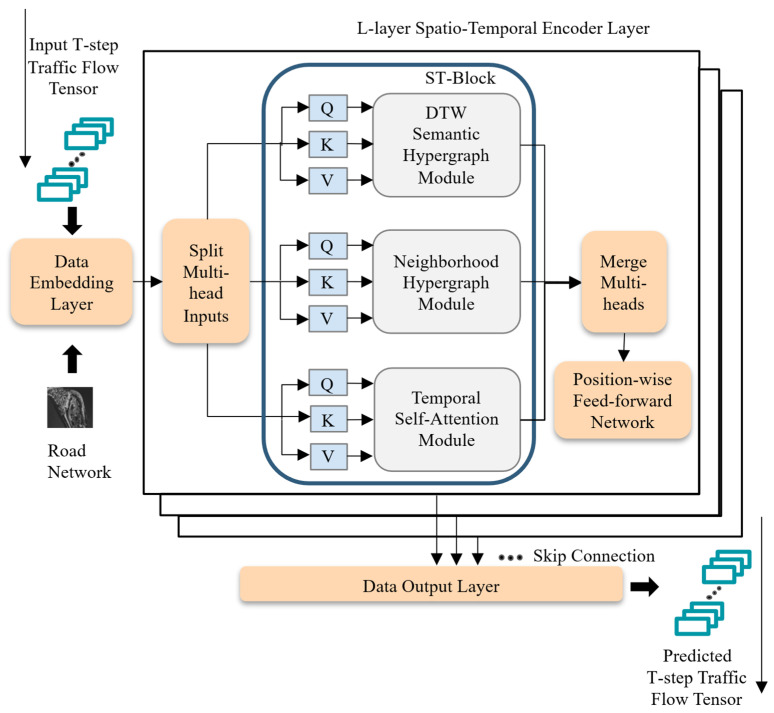
Model structure diagram.

The overall architecture of the proposed HGSTIN is illustrated in Figure 2. This section sequentially introduces the spatio-temporal positional encoding, data embedding layer, Spatio-Temporal Feature Extraction Block (ST-Block), adaptive feature fusion module, temporal gradient consistency loss function, and data output layer, systematically presenting the modeling and prediction process for complex traffic flow features.

The ST-Block integrates three sub-modules: the Temporal Self-Attention module, the Neighborhood Hypergraph module, and the DTW Semantic Hypergraph module, which are designed to capture temporal dependencies, local spatial correlations, and global spatial semantic correlations, respectively. Through a triple-path attention mechanism, the ST-Block enables efficient extraction and fusion of multi-level spatio-temporal features within a unified framework. HGSTIN employs a multi-layer stacked ST-Block structure, which progressively abstracts and fuses features across layers to enhance the representation of complex spatio-temporal patterns, providing discriminative features for traffic forecasting.

### 3.1. Spatio-Temporal Positional Encoding

(1) Temporal Positional Encoding

To endow the model with temporal awareness, a three-level temporal encoding mechanism is designed. First, absolute position information is introduced via sine and cosine functions to enable the identification of specific time points. Before the sequence is fed into the temporal module, absolute positional encodings are generated as follows:

(6) $$E_{tpe}\left(t,2d\right)=\sin\left(t/10000^{2d/d_{model}}\right)$$

(7) $$E_{tpe}\left(t,2d+1\right)=\cos\left(t/10000^{2d/d_{model}}\right)$$

where t is the time index, $d_{model}$ is the hidden dimension, and each vector has dimension d.

Subsequently, trainable periodic embeddings are incorporated. The Time-of-Day embedding maps normalized minutes to integer indices via a trainable table $E_{day}\;{\in R}^{1440\times d_{model}}$, while the Day-of-Week embedding maps the days of the week to learnable vectors $E_{week}\;{\in R}^{7\times d_{model}}$. Finally, the timestamp information is concatenated with the dynamic data to form an input tensor of dimension B×T×N×(F+8), allowing for joint modeling of numerical and temporal semantic features.

(2) Spatial Positional Encoding

A normalized Laplacian matrix is constructed to represent the road network topology:

(8) $$\Delta =I-D^{-1/2}AD^{-1/2}$$

where $A\;{\in R}^{N\times N}$ is the adjacency matrix, I is the identity matrix, and D is the degree matrix. Each row of the Laplacian matrix serves as a static node feature, which is projected through a learnable linear mapping into spatial positional embeddings $E_{spe}\;{\in R}^{N\times d_{model}}$ to retain global topological information while reducing dimensionality.

### 3.2. Data Embedding Layer

The data embedding layer serves as the core preprocessing module, converting low-dimensional traffic features into high-dimensional representations. A feed-forward network (TokenEmbedding) maps multi-periodic features into a high-dimensional space, which are then concatenated along the temporal dimension and fused with other spatio-temporal features:

(9) $$H^{\left(0\right)}=TokenEmbedding\left(\psi_{\mathrm{data}}\right)\in R^{B\times N\times F\times \left(T_{r}+d*T_{k}+w*T_{k}\right)\times d_{model}}$$

where $d_{model}$ denotes the dimension of the target high-dimensional space, and $\psi_{\mathrm{data}}$ represents the input traffic data.

The mapped feature $H^{\left(0\right)}$ is then aggregated with the fixed sine-cosine temporal positional encodings, learnable daily/weekly periodic embeddings, and spatial positional embeddings to form the final spatio-temporal embedding representation. This fusion process, termed spatio-temporal encoding integration, utilizes an additive operation to combine the up-sampled features with the positional encodings, resulting in an enhanced spatio-temporal embedding:

(10) $$H_{emb}=H^{\left(0\right)}+E_{\mathrm{spe}}+E_{\mathrm{tpe}}+E_{day}+E_{week}$$

This design reduces computational overhead through parameter sharing while preserving the independence of spatio-temporal features.

### 3.3. Temporal Self-Attention Module

The primary function of the Temporal Self-Attention module is to capture cross-time-step dependencies within the traffic flow data, such as the persistent influence of morning peak traffic volume on subsequent periods. To better handle non-stationary time series and systematically model the evolutionary dynamics of short-term traffic flow, this module integrates the Dynamic Tanh (DyT) mechanism with a multi-head self-attention architecture. Specifically, the DyT mechanism performs adaptive nonlinear scaling to precisely capture temporal gradients, particularly during periods of significant fluctuation. The multi-head attention components then process these dynamically optimized features to extract periodicity and trend patterns across different time scales in parallel, thereby establishing a rigorous representation of long-range temporal dependencies. To execute this process, the module introduces a three-step computational workflow: Dynamic Nonlinear Transformation, Multi-Head Self-Attention Calculation, and Residual Connection.

(1) DyT Dynamic Nonlinear Transformation Mechanism

Prior to the attention computation, this module introduces the Dynamic Tanh (DyT) nonlinear adaptation mechanism. Both the subsequent Neighborhood Hypergraph module and the DTW Semantic Hypergraph module follow a unified computational architecture of “dynamic adaptation followed by feature mapping,” where all modules share the output features $H_{emb}$ from the data embedding layer as the raw input. Distinct from traditional static layer normalization, DyT leverages the nonlinear saturation characteristics of the hyperbolic tangent function (tanh) and learnable dynamic parameters to perform adaptive nonlinear scaling and bias adjustment on the features. The calculation is formulated as follows:

(11) $$\tilde{H}=\gamma \cdot tanh\left(\alpha H_{emb}\right)+\beta$$

where $\tilde{H}$ denotes the intermediate feature matrix after dynamic adjustment; α, γ, and β are learnable parameters utilized to dynamically adjust the slope, feature magnitude, and bias center of the nonlinear activation function, respectively.

(2) Multi-Head Self-Attention Calculation

In the current module and subsequent modules involving spatial attention, the dynamically optimized features $\tilde{H}$ are mapped into Query (Q), Key (K), and Value (V) vectors. These vectors are generated in parallel via convolutional operations:

(12) $$Q_{n}^{\left(u\right)}=W_{Q}\tilde{H},K_{n}^{\left(u\right)}=W_{K}\tilde{H},V_{n}^{\left(u\right)}=W_{V}\tilde{H}$$

where $Q_{n}^{\left(u\right)},K_{n}^{\left(u\right)},V_{n}^{\left(u\right)}\in R^{T\times d_{model}}$; $W_{Q}$, $W_{K}$, and $W_{V}$ are learnable parameters, n denotes the node index, and u signifies the temporal self-attention module. The multi-head self-attention distribution is calculated as follows:

(13) $$Attention\left(Q_{n}^{\left(u\right)},K_{n}^{\left(u\right)},V_{n}^{\left(u\right)}\right)=Softmax\left(\frac{{Q_{n}^{\left(u\right)}{(K}_{n}^{\left(u\right)})}^{T}}{\sqrt{d_{k}}}\right)V_{n}^{\left(u\right)}$$

where $d_{k}$ is the dimension of the key vector. Through the multi-head mechanism, the module can capture periodicity and trends across various time scales in parallel, yielding richer temporal dependency features.

(3) Residual Connection

Following the DyT dynamic adjustment and temporal self-attention calculation, the module employs a residual connection to fuse the input and output features. This design prevents the gradient vanishing problem caused by deep network stacking, thereby ensuring training stability. The final output temporal features are calculated as follows:

(14) $$H_{time}={H_{time}}^{\left(l-1\right)}+Dropout\left(Attention\left(Q_{n}^{\left(u\right)},K_{n}^{\left(u\right)},V_{n}^{\left(u\right)}\right)\right)$$

where $H_{time}\in R^{T\times d_{model}}$ is the output temporal feature matrix, and ${H_{time}}^{\left(l-1\right)}$ represents the temporal feature representation from the previous layer, which corresponds to the data embedding layer output $H_{emb}$ for the first layer; and $Attention\left(\cdot \right)$ denotes the multi-head attention calculation result optimized by the DyT input.

### 3.4. Neighborhood Hypergraph Module

(1) Spatial Self-Attention Calculation and Hypergraph Mask

The Neighborhood Hypergraph module is designed to capture local spatial dependencies among nodes within the traffic network. By modeling local traffic congestion propagation through the HGNN, it facilitates the efficient modeling of both global and local spatial features.

Based on geographic topological relations, a geographic adjacency matrix $A_{geo}\in R^{N\times N}$ is predefined to aggregate physical neighborhood information, where N is the number of traffic nodes. Each node and its first-order neighbors are defined as a hyperedge $\varepsilon_{geo}$, generating an incidence matrix $H_{geo}\in {\left\{0,1\right\}}^{N\times |\varepsilon_{geo}|}$ that covers the local connectivity structure. The specific formula is as follows:

(15) $$H_{geo}\left(i,j\right)=\left\{\begin{array}{c} 1,\;\;\;\;\;\;\;\;\;\;\;\;\mathrm{if}\;S_{geo}\left(i,j\right)<\delta_{geo}\\ \;\;0,\;\;\;\;\;\;\;\;\;\;\;\;\;\;\;\;\;\;\;\;\mathrm{otherwise} \end{array}\right.$$

where $S_{geo}\left(i,j\right)$ denotes the shortest path hop count between node i and node j calculated based on $A_{geo}$, that is, the minimum number of road segments required to connect two sensor nodes in the road network topology; $\delta_{geo}$ represents the predefined hop-count truncation threshold, which is utilized to control the receptive field range of the physical neighborhood.

The input feature $\tilde{H}$ generates $Q_{n}^{\left(s\right)}$ and $K_{n}^{\left(s\right)}$ in the same manner as described in Section 3.3. These vectors are utilized to calculate spatial attention scores, and the neighborhood hypergraph structure is introduced as a mask to shield the attention scores of unconnected node pairs:

(16) $$Attention\left(Q_{n}^{\left(s\right)},K_{n}^{\left(s\right)}\right)=Softmax\left(\frac{{Q_{n}^{\left(s\right)}{(K}_{n}^{\left(s\right)})}^{T}}{\sqrt{d_{k}}}+M\right)$$

where M is the mask matrix. If a node pair (i,j) has no connection in the hypergraph, $M_{ij}=-\infty$, ensuring that the weight of this node pair becomes zero after normalization.

The Q, K, and V vectors for both the Neighborhood Hypergraph and DTW Semantic Hypergraph branches are derived from the outputs of the Temporal Self-Attention module. However, each branch uses independent projection layers for Q, K, and V to transform these temporal embeddings into branch-specific spatial attention inputs. This allows the Neighborhood branch to focus on local adjacency while the DTW Semantic branch captures long-range pattern similarity, without sharing parameters across branches.

(2) Dual-Branch Calculation for $V_{n}^{\left(s\right)}$

A dual-branch structure is adopted for the calculation of $V_{n}^{\left(s\right)}$. Two sets of linear transformations are executed in parallel on input feature to generate the value vector $V_{n1}^{\left(s\right)}$ for the spatial self-attention branch and the value vector $V_{n2}^{\left(s\right)}$ for the hypergraph neural network branch. These two branches separately model spatial features at different levels, which are subsequently integrated through a fusion module.

The first path is the spatial self-attention branch, which multiplies $V_{n1}^{\left(s\right)}$ by the attention scores to obtain the node feature output based on the spatial self-attention mechanism:

(17) $$H_{attn}=Attention\left(Q_{n}^{\left(s\right)},K_{n}^{\left(s\right)}\right)\cdot V_{n1}^{\left(s\right)}$$

**Figure 3:**
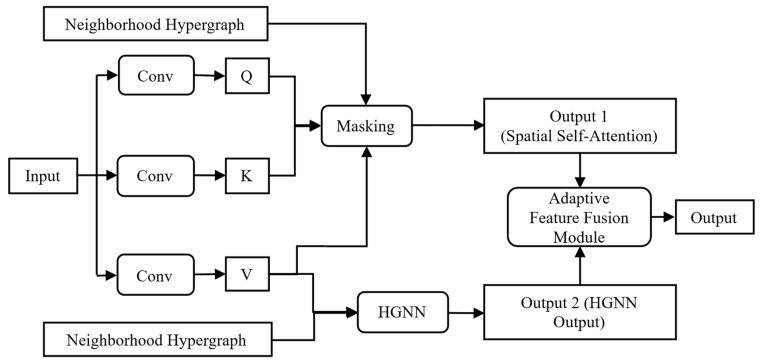
Structure diagram of neighborhood hypergraph module.

The second path is the hypergraph neural network (HGNN) branch, which combines $V_{n2}^{\left(s\right)}$ with the neighborhood hypergraph structure and inputs it into the HGNN. This achieves high-order information passing and aggregation between nodes through hypergraph convolution, yielding the following output:

(18) $$H_{hgnn}=\sigma \left(LV_{n2}^{\left(s\right)}\right)$$

where σ is the activation function, and L is the normalized hypergraph Laplacian matrix. The specific calculations are as follows:

(19) $$D_{v}=diag\left(\sum_{j}H_{geo}\left(i,j\right)\right)$$

(20) $$D_{e}=diag\left(\sum_{i}H_{geo}\left(i,j\right)\right)$$

(21) $$L=D_{v}^{-\frac{1}{2}}H_{geo}W_{geo}D_{e}^{-1}{H_{geo}}^{T}D_{v}^{-\frac{1}{2}}$$

where $D_{v}$ is the node degree matrix, and $D_{e}$ is the hyperedge degree matrix. The hyperedge weight matrix is $W_{geo}\in R^{N\times N}$. The outputs from the two branches, $H_{attn}$ and $H_{hgnn}$, will be combined through the adaptive feature fusion module to fully leverage the complementary advantages of both types of spatial features. The fusion module will be detailed in Section 3.6, and the structure of the Neighborhood Hypergraph module is illustrated in Figure 3.

### 3.5. DTW Semantic Hypergraph Module

This study further designs a dynamic semantic hypergraph structure based on Dynamic Time Warping (DTW) similarity to generate high-order spatial dependencies, effectively supplementing long-range spatial correlations. The DTW Semantic Hypergraph module captures the global collaborative variations of distant nodes by modeling the similarity of traffic patterns. Its architecture is similar to the Neighborhood Hypergraph module, employing a combination of Transformer-based spatial self-attention and HGNN for spatial feature modeling. The core difference lies in the generation of the similarity matrix and hyperedges.

This module relies on a precomputed DTW distance matrix $A_{dtw}$ to measure the temporal-pattern distance between traffic nodes. For each node, the DTW distances between this node and all other nodes are sorted in ascending order. Then, the top k nodes with the smallest DTW distances are selected as the most semantically similar nodes and assigned to the same semantic hyperedge $\varepsilon_{dtw}$. In this way, the semantic hypergraph incidence matrix $H_{dtw}\in {\left\{0,1\right\}}^{N\times |\varepsilon_{dtw}|}$ is generated as follows:

(22) $$H_{dtw}\left(i,j\right)=\left\{\begin{array}{c} 1,\;\;\;\;\;if\;i\in S_{dtw}\left(j\right)\\ 0,\;\;\;\;\;\;\;\;\;\;\;\mathrm{otherwise} \end{array}\right.$$

where $H_{dtw}\left(i,j\right)$ determines whether node i is included in the semantic hyperedge centered on node *j*, and $S_{dtw}\left(j\right)$ denotes the set of neighboring nodes most semantically similar to node *j* after sorting the DTW distances. The number of selected semantic neighbors is denoted as $\delta_{dtw}$.

For each traffic sensor, $\delta_{dtw}$ most semantically similar neighbors are selected to form a semantic hyperedge. One semantic hyperedge is generated per sensor, so the total number of semantic hyperedges is fixed as N, where N denotes the number of traffic sensors. The DTW-based semantic hypergraph is precomputed and remains fixed during training and inference. The dynamic property of the model is reflected only in input-dependent attention weights and adaptive fusion, rather than online updates of the hypergraph topology.

The innovation of this approach lies in the ability of the semantic hypergraph to capture long-distance spatio-temporal dependencies in traffic flow data, particularly the similarities between cross-regional and cross-temporal nodes. Unlike traditional graph structures based on physical adjacency, this method can model more complex dependencies by capturing global traffic pattern similarities. The fundamental difference between the two types of spatial modules is that this module utilizes the semantic incidence matrix $H_{dtw}$ instead of the physical incidence matrix $H_{geo}$ to model high-order semantic dependencies between remote nodes. Therefore, the structural diagram of this module is not repeated here.

### 3.6. Adaptive Feature Fusion Module

This module serves as the internal structure for both the Neighborhood Hypergraph and DTW Semantic Hypergraph modules, enhancing their capability to represent complex spatial dependencies. The module first concatenates the two types of spatial features within the module along the feature dimension, then reduces the concatenated feature dimension to 2 through a linear layer to calculate weights for weighted fusion. Since both modules utilize the same adaptive feature fusion mechanism, the fusion process is described here using the Neighborhood Hypergraph module as an example.

(1) Input Features and Dynamic Weight Calculation

The inputs to the adaptive feature fusion module come from the two branches within the hypergraph module; for the Neighborhood Hypergraph module, these are $H_{attn}$ and $H_{hgnn}$. First, $H_{attn}$ and $H_{hgnn}$ are concatenated along the feature dimension. The concatenated feature tensor then generates a 2D weight vector p via a linear layer:

(23) $$p=LeakyReLU\left(W_{geo}\left[H_{attn}∥H_{hgnn}\right]\right)$$

where the weight matrix $W_{geo}\in R^{d_{model}\times 1}$ represents learnable parameters. The weight tensor is generated by normalizing along the last dimension:

(24) $$g=Softmax\left(p\right)$$

where $g\in R^{B\times T\times N\times 2}$ is the normalized weight matrix, consisting of two parts denoted as $g=[g_{1},g_{2}]$. Here, $g_{1}\in R^{B\times T\times N\times 1}$ represents the weight matrix for the spatial self-attention branch, and $g_{2}\in R^{B\times T\times N\times 1}$ represents the weight matrix for the HGNN branch. Each row of the weight matrix corresponds to a node’s weight vector $\left[g_{i1},g_{i2}\right]$, satisfying $g_{i1}+g_{i2}=1$, which allows for dynamic weight allocation according to specific traffic flow scenarios.

(2) Feature Fusion

The fused features are calculated through a weighted sum:

(25) $$H_{geo}^{\prime }=g_{1}⊙H_{attn}+g_{2}⊙H_{hgnn}$$

where ⊙ denotes element-wise multiplication for feature weighting. The output is $H_{geo}^{\prime }\in R^{B\times T\times N\times d_{model}}$.

### 3.7. Temporal Gradient Consistency Loss Function

To improve the robustness and temporal rationality of the prediction model, this study designs a temporal gradient loss function based on a dual-objective joint optimization strategy. First, the Huber Loss function is employed; it uses squared loss to accelerate convergence when the prediction error is small and automatically switches to linear loss when the error exceeds a preset threshold to suppress the influence of noise in traffic data. Its mathematical expression is as follows:

(26) $$L_{H}=\left\{\begin{array}{c} 0.5{\left(y-\hat{y}\right)}^{2},\;\;\;\;\;\;\;\;\;\;\;\;\;\;\;\;\;\;\left|y-\hat{y}\right|\leq \delta \\ \delta \left(\left|y-\hat{y}\right|-0.5\delta \right)\;\;\;\;\;\;\;\;\;\;\;\;\;otherwise \end{array}\right.$$

where δ is the preset threshold controlling the smoothing interval of the loss function, y is the ground truth, and $\hat{y}$ is the predicted value. By utilizing the quadratic form of MSE for small errors and switching to the linear form of MAE for larger errors, the model can rapidly fit the normal data distribution while effectively filtering out abnormal signals such as sensor failures.

Building upon this, the Temporal Gradient Loss function is incorporated:

(27) $$L_{G}=\frac{1}{Q-1}\sum_{t=1}^{Q-1}\left|\nabla {\hat{y}}_{t}-\nabla y_{t}\right|$$

where Q is the length of the predicted sequence, $\nabla {\hat{y}}_{t}={\hat{y}}_{t+1}-{\hat{y}}_{t}$ is the temporal gradient of the predicted sequence, and $\nabla y_{t}=y_{t+1}-y_{t}$ is the temporal gradient of the ground truth sequence. This loss forces the model to learn the evolution laws of the traffic state by comparing step-by-step changes, constraining the predicted sequence’s trend to be consistent with the real data. The final loss function is a weighted combination of the two:

(28) $$L=L_{H}+{\omega L}_{G}$$

where ω is a balancing factor that controls the intensity of the gradient constraint. ω controls the relative contribution of the temporal gradient consistency loss: a very small value weakens the temporal trend constraint, while a very large value may overemphasize smoothness and slightly sacrifice point-wise prediction accuracy. Therefore, ω was chosen to balance prediction accuracy and temporal trend consistency. Experiments demonstrate that this phased strategy enables the model to generate smooth results consistent with traffic dynamic characteristics while ensuring prediction accuracy.

### 3.8. Data Output Layer

The computed features are integrated via concatenation, with the resulting spatio-temporal features denoted as $H_{space}$. In this model, the output layer adopts a structure that combines a feed-forward neural network (FFN) with two layers of one-dimensional (1D) convolutional networks.

The features first pass through the FFN for nonlinear transformation and enhancement. Specifically, the FFN performs a linear transformation through fully connected layers and introduces nonlinearity via the ReLU activation function, thereby strengthening the model’s capacity to represent complex spatio-temporal dependencies. Subsequently, the processed features are fed into two 1D convolutional layers, denoted as ${Conv}_{1}$ and ${Conv}_{2}$, for dimensionality reduction. In the specific implementation, features are first fused and enhanced using multi-layer encoders and skip-connection convolutions, followed by dimensionality reduction via ${Conv}_{1}$ and ${Conv}_{2}$. ${Conv}_{1}$ maps the temporal dimension from the input window length T to the prediction window length $T_{out}$, while ${Conv}_{2}$ reduces the feature dimension to the target output dimension. The final output tensor is $\hat{Y}\in R^{B\times T_{out}\times N\times D_{out}}$, where $D_{out}$ is the number of output variables. The calculation for the output layer is expressed as follows:

(29) $$\hat{Y}={Conv}_{2}(ReLU({Conv}_{1}\left(ReLU\left(skip\right)\right)))$$

where skip represents the fused feature tensor and ReLU is the activation function. Through this output layer design, the model effectively transforms multi-level, fused spatio-temporal features into specific traffic flow predictions, meeting the requirements of downstream traffic management and decision-making. The parameters of the output layer are jointly optimized with the loss function against ground-truth observations to ensure the accuracy and temporal rationality of the results.

## 4. Experimental Analysis

### 4.1. Data Sources

The traffic flow data utilized in this study were obtained from the Performance Measurement System (PeMS) of the California Department of Transportation. Two publicly available datasets, PEMS04 and PEMS08, were partitioned chronologically into training, validation, and testing sets with a ratio of 60%, 20%, and 20%, respectively. Z-Score normalization was applied to the traffic flow data as a preprocessing step. The basic information of the datasets is summarized in Table 1.

### 4.2. Baseline Models

Considering structural similarity, theoretical frontiers, and result effectiveness, seven classic traffic forecasting models were selected as baselines for comparison:

(1) SVR [12]: Support Vector Regression.
(2) LSTM [13]: Long Short-Term Memory network, an enhanced version of Recurrent Neural Networks (RNNs) that utilizes gating mechanisms to effectively address the gradient vanishing problem.
(3) DCRNN [14]: Diffusion Convolutional Recurrent Neural Network.
(4) STGCN [4]: Spatio-Temporal Graph Convolutional Network, which captures both spatial correlations between nodes and dynamic temporal features through a spatio-temporal convolutional structure.
(5) Graph WaveNet [15]: A model that integrates graph convolutions with dilated convolutions, utilizing multi-scale filter banks to adaptively capture spatio-temporal dependencies.
(6) ASTGCN [16]: Attention-based Spatio-Temporal Graph Convolutional Network, which employs three parallel branches to process the three periodic patterns of the data.
(7) ASTGNN [17]: Attention-based Spatio-Temporal Graph Neural Network, which features a trend-aware self-attention module combined with dynamic graph convolutions to learn spatio-temporal heterogeneous features.

### 4.3. Experimental Settings

The model uses 1 h of historical data ($T_{r}$ = 12) to predict traffic flow for the subsequent hour ($T_{k}$ = 12). Experiments were conducted on a workstation equipped with an NVIDIA RTX 4090D GPU (24 GB VRAM).

The software platform was built using Python 3.8.19 and the PyTorch 2.0.1 deep learning framework, with the Adam optimizer employed for training. The hidden dimension d was set to 256, and the number of spatio-temporal layers l was set to 6. The number of attention heads for the Temporal Self-Attention, Neighborhood Hypergraph, and DTW Semantic Hypergraph modules was set to 2. Regarding the overall configuration, the initial learning rate was 0.001 and the batch size was 16. Each experiment was repeated 10 times, with 200 iterations (epochs) per run.

### 4.4. Evaluation Metrics

To evaluate model performance, the following three metrics were utilized:

(1) Mean Absolute Error (MAE). The formula is expressed as follows:

(30) $$MAE=\frac{1}{n}\sum_{i=1}^{n}\left|y_{i}-{\hat{y}}_{i}\right|$$

(2) Root Mean Square Error (RMSE). The formula is expressed as follows:

(31) $$RMSE=\sqrt{\frac{1}{n}\sum_{i=1}^{n}{\left(y_{i}-{\hat{y}}_{i}\right)}^{2}}$$

(3) Mean Absolute Percentage Error (MAPE). The formula is expressed as follows:

(32) $$MAPE=\frac{1}{n}\sum_{i=1}^{n}\left|\frac{y_{i}-{\hat{y}}_{1}}{y_{i}}\right|$$

In these equations, n denotes the number of samples, $y_{i}$ represents the observed value of the i-th sample, and ${\hat{y}}_{i}$ represents the corresponding predicted value.

### 4.5. Experimental Results and Analysis

#### 4.5.1. Comparison with Baseline Models

The prediction results for the next 1 h (comprising 12 steps, with a 5 min interval per step) are presented in Table 2. For each dataset, the optimal results are highlighted in bold, while the second-best results are underlined.

(1) The proposed HGSTIN demonstrates a clear overall performance advantage, significantly outperforming all baseline models across all evaluation metrics on both the PEMS04 and PEMS08 datasets. On the PEMS04 dataset, compared to the second-best model ASTGNN, it reduces the MAE by 0.978%, RMSE by 3.224%, and MAPE by 2.870%. On the PEMS08 dataset, compared to the optimal baseline Graph WaveNet, it reduces the MAE by 9.327%, RMSE by 1.006%, and MAPE by 4.884%.

**Figure 4:**
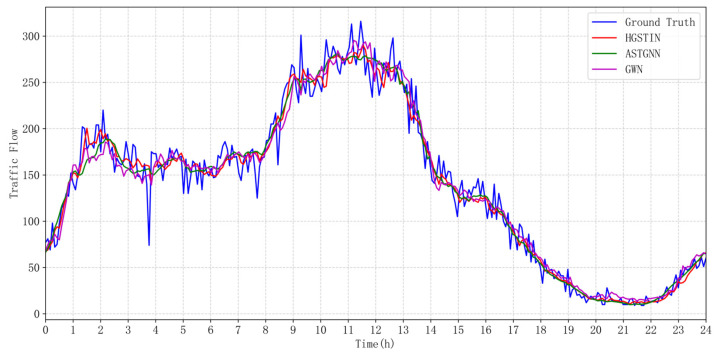
Prediction curve of a certain node in the PEMS04 dataset.

**Figure 5:**
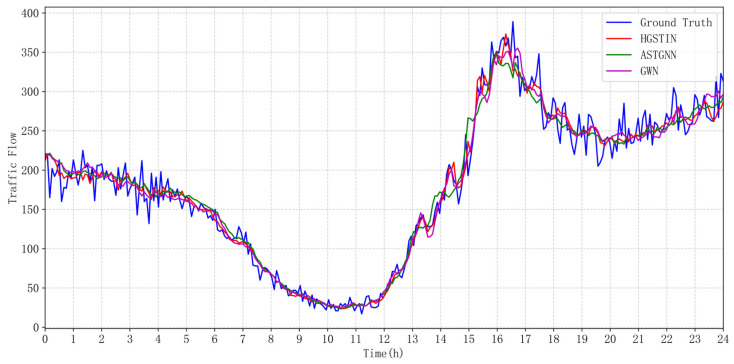
Prediction curve of a certain node in the PEMS08 dataset.

A continuous 24 h traffic flow sequence from a randomly selected station in each dataset was extracted to plot the prediction curves, as shown in Figure 4 and Figure 5. These visualizations provide real-world traffic prediction case studies for comparing HGSTIN with representative baseline models. It can be observed that HGSTIN follows the ground-truth traffic variations more closely, while maintaining consistent trends during peak periods and fluctuating intervals.

(2) The experimental datasets utilized in this study are derived from actual traffic monitoring systems, intrinsically containing sudden incidents and complex fluctuations typical of real-world traffic scenarios. The data encompass factors such as traffic accidents, adverse weather, holiday travel peaks, and road construction, which cause severe fluctuations in traffic flow during different periods. Therefore, our experiments have been validated on complex, dynamic, and non-linear real-world data, eliminating the need to introduce additional simulations for sudden incidents or complex fluctuations. Among the traditional models, SVR and LSTM exhibit the poorest prediction performance. In contrast, graph structure-based models, including DCRNN, STGCN, and Graph WaveNet, demonstrate significantly improved performance by incorporating spatial feature modeling. However, these models remain constrained by static adjacency matrices or fixed convolutional kernels, failing to fully capture dynamic spatio-temporal dependencies. STGCN and ASTGCN rely on static graph structures, resulting in MAPE values on PEMS04 that are 18.654% and 29.242% higher than those of the proposed HGSTIN model, respectively, further highlighting the necessity of dynamic spatial modeling.

(3) ASTGNN is the best-performing attention-based model, notably outperforming GCN- and TCN-based models. The primary reason for this performance gap is that as network depth increases, GCN and TCN models are prone to the over-smoothing phenomenon, making it difficult to effectively capture long-term temporal dependencies. ASTGCN employs three independent branches to model the current, daily, and weekly traffic flow patterns, using 1D CNNs to extract temporal features and GCNs to capture spatial structures. However, convolutional kernels suffer from limited receptive fields, making it challenging for the model to model long-term temporal dependencies. Furthermore, independently modeling each periodic branch reveals a deficiency in mining the intrinsic correlations among different periodic patterns. Consequently, its prediction performance remains inferior to the proposed HGSTIN model.

(4) By explicitly fusing three types of traffic features and three periodic data sources to construct high-dimensional input features, HGSTIN can fully mine the correlations between different periodic patterns. The introduction of auxiliary features enables the model to more accurately identify changes in spatial features, improving its prediction accuracy in complex traffic environments. Regarding temporal modeling, HGSTIN effectively captures periodic and trend variations in time series by utilizing a Transformer architecture with an incorporated DyT mechanism, enhancing the model’s ability to represent long-term temporal dependencies. For spatial modeling, HGSTIN adopts a dual mechanism consisting of a dynamic semantic hypergraph and a neighborhood hypergraph. Combined with a masking operation to capture global spatial similarities, the multi-head self-attention mechanism, and the adaptive feature fusion module, it effectively models the complex dynamic spatio-temporal dependencies of the data. Furthermore, HGSTIN incorporates a temporal gradient consistency loss function that constrains the temporal gradients of the predicted and ground truth sequences, effectively suppressing abnormal fluctuations and overfitting noise. This ensures that the model not only outperforms existing methods in numerical accuracy but also generates smoother prediction results consistent with the actual evolution laws of traffic flow.

#### 4.5.2. Effect Analysis at Different Prediction Time Steps

Multi-step traffic flow prediction experiments for 3 steps (15 min) and 6 steps (30 min) ahead were conducted on both datasets. Combined with the 12-step ahead prediction results from the previous section, the performance changes of each model as the prediction time step increases are compared, as shown in Table 3 and Table 4. Since the previous experimental results indicated that SVR and LSTM performed poorly in the 1-h ahead prediction, their prediction results for different time steps are omitted here.

Experimental data demonstrate that while the difficulty of the prediction task increases as the prediction horizon extends, the HGSTIN model exhibits the most minimal performance decay across various prediction intervals. This validates the superiority of the proposed model in multi-step forecasting scenarios.

**Figure 6:**
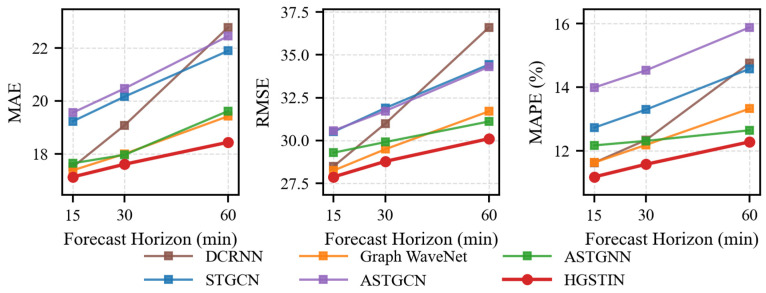
Trends in multi-step prediction accuracy on the PEMS04 datasets.

**Figure 7:**
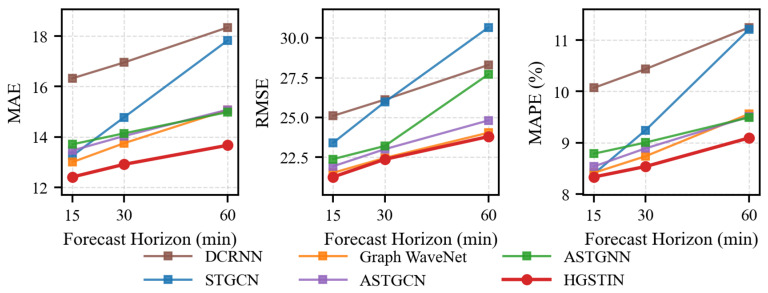
Trends in multi-step prediction accuracy on the PEMS08 datasets.

To further characterize the nonlinear cumulative evolution of prediction errors with increasing forecasting horizons, Figure 6 and Figure 7 illustrate the complete trends of multi-step prediction performance from 15 to 60 min, based on the tabulated results and the 12-step-ahead prediction metrics obtained in the previous section.

As shown in Figure 6 and Figure 7, the prediction errors of most models increase as the forecasting horizon extends from 15 min to 60 min, indicating that long-horizon traffic prediction becomes more challenging due to the accumulation of temporal uncertainty. However, the error curves of HGSTIN remain consistently lower than those of the baseline models in terms of MAE, RMSE, and MAPE on both datasets. In particular, the red curves corresponding to HGSTIN exhibit a relatively smooth upward trend, suggesting that the proposed model suffers less error accumulation when the prediction horizon increases.

As an RNN-based model, DCRNN shows limitations in capturing long-term temporal dependencies, and its prediction errors increase noticeably in the 60 min forecasting task. STGCN and Graph WaveNet utilize convolutional or temporal convolutional structures to extract temporal features, but their performance may still be affected by the limited receptive field when modeling long-range temporal evolution. ASTGCN relies heavily on predefined spatial graph structures, which restricts its ability to adaptively capture dynamic spatial dependencies. Although ASTGNN improves spatio-temporal modeling through attention mechanisms and dynamic graph learning, it does not explicitly construct high-order neighborhood and semantic hypergraph relationships. Therefore, its performance is still inferior to HGSTIN in most long-horizon forecasting cases.

By integrating dynamic temporal modeling with both neighborhood and DTW semantic hypergraph modules, HGSTIN can simultaneously capture local spatial adjacency, long-range semantic similarity, and multi-scale temporal dependencies. In addition, the incorporation of auxiliary features and multi-period inputs further enhances the model’s perception of traffic periodicity and complex spatio-temporal variations. Consequently, HGSTIN maintains more stable and superior performance across different prediction horizons, demonstrating stronger robustness in multi-step traffic forecasting scenarios.

#### 4.5.3. Ablation Study

To verify the impact of individual modules on model performance, ablation experiments were conducted on both datasets. Several model variants were constructed and named as follows to facilitate a comparative analysis with the complete HGSTIN model:

(1) w/o Multi-feature and Multi-period Input: A version of HGSTIN that excludes multi-feature and multi-period modeling. This variant uses only raw traffic flow data as input and omits periodic features such as recent, daily, and weekly patterns, thus failing to fully mine the periodic regularities of traffic flow.
(2) w/o Neighborhood Hypergraph: A version of HGSTIN that retains only the semantic hypergraph branch, utilizing solely the DTW-similarity-based semantic hypergraph for spatial modeling.
(3) w/o Semantic Hypergraph: A version of HGSTIN that retains only the neighborhood hypergraph branch, utilizing solely the physical-adjacency-based neighborhood hypergraph for spatial modeling.
(4) w/o Adaptive Fusion: A version of HGSTIN without the adaptive feature fusion module; instead, the features are combined through simple addition.
(5) w/o Temporal Gradient Loss: A version of HGSTIN that utilizes only the Huber loss as the objective function.

The results of the ablation experiments are summarized in Table 5.

Based on the analysis of the experimental results, the following conclusions can be drawn:

(1) The semantic hypergraph structure plays a significant role in modeling functional similarity across nodes. Upon removing this structure, both the MAE and MAPE metrics across the two datasets increased by more than 5%. This indicates that the semantic hypergraph effectively mitigates spatial heterogeneity between sensors and enhances the model’s generalization capability.
(2) As a key module for capturing local spatial structures, the neighborhood hypergraph has the most significant impact on performance enhancement. The removal of this structure led to the largest increases in all evaluation metrics for both PEMS08 and PEMS04, demonstrating that this module plays a core role in expanding the spatial receptive field and strengthening spatial dependency modeling.
(3) The multi-feature and multi-period input module assists the model in representing repetitive temporal patterns in urban traffic. After removing this module, the prediction performance on both datasets declined consistently but moderately. This indicates that the multi-feature and multi-period input provides useful supplementary temporal information, but it is not the dominant source of the overall performance improvement. The main performance gain of HGSTIN is more closely related to the dual hypergraph spatial modeling and adaptive fusion mechanism. Therefore, the multi-feature and multi-period input can be regarded as a complementary temporal prior that enhances the robustness of the learned spatio-temporal representations.
(4) The adaptive gating strategy introduced in the fusion mechanism effectively enhances the complementarity between different features. Although the performance degradation after its removal was relatively small, it showed a consistent downward trend, suggesting that this mechanism positively contributes to the synergistic modeling of multi-source information.
(5) The temporal gradient consistency loss function guides the model to focus on the evolution trends of traffic states, increasing sensitivity to volatile data. After ablating this loss term, the MAPE metric rose significantly, highlighting its importance in trend modeling, particularly in medium- and long-term prediction scenarios where its effect is more pronounced.

In summary, the ablation results on PEMS04 and PEMS08 verify the effectiveness of the main components of HGSTIN. Among them, the dual hypergraph spatial modeling modules and the adaptive fusion mechanism contribute more substantially to the overall performance, while the multi-feature and multi-period input provides complementary temporal information for improving prediction robustness.

## 5. Conclusions

To meet the prediction requirements for traffic flow in complex road networks, this paper proposes the HGSTIN model based on a hypergraph spatio-temporal interaction network. Validated on two real-world traffic flow datasets, the following primary conclusions are reached:

(1) This study developed the HGSTIN model, which integrates multi-feature multi-period inputs, dynamic semantic and neighborhood hypergraph spatial modeling, adaptive feature fusion, and temporal gradient loss constraints. Experimental results demonstrate that HGSTIN outperforms existing mainstream baseline models in key evaluation metrics such as MAE, RMSE, and MAPE on the PEMS04 and PEMS08 datasets. Compared to the optimal baseline models, HGSTIN demonstrates superior performance in short-term traffic flow prediction tasks.
(2) Compared to ASTGCN, which models periodic data independently, HGSTIN explicitly fuses auxiliary features—including proximity, daily periodicity, and weekly periodicity—with spatio-temporal positional encoding to deeply mine multi-scale temporal similarities and static spatial features. Results show that HGSTIN achieves average improvements of 13.618%, 8.158%, and 13.485% over ASTGCN across all metrics, significantly enhancing the model’s ability to capture periodic patterns. Compared to GCN- or TCN-based models like ASTGNN, STGCN, and Graph WaveNet, HGSTIN utilizes a dual-spatial modeling mechanism (dynamic semantic and neighborhood hypergraphs) and masking operations to capture spatial correlations of local neighborhoods and remote nodes respectively. Combined with a multi-head self-attention mechanism, this enhances the model’s capability to handle complex spatial dependencies.
(3) HGSTIN incorporates an adaptive feature fusion module within the spatial feature component to achieve adaptive integration of the dual-branch outputs. Furthermore, a temporal gradient consistency loss function was designed, utilizing a joint optimization strategy of Huber loss and temporal gradient loss. This constrains the temporal variation trends of the predicted sequences and effectively suppresses abnormal fluctuations and overfitting noise. Consequently, the model not only surpasses existing methods in numerical accuracy but also generates prediction results that more closely align with the actual evolution laws of traffic flow.

The performance enhancements demonstrated by the HGSTIN model hold significant application value for practical traffic management and decision-support systems. Improving the accuracy of traffic flow prediction can substantially enhance the scheduling efficiency of traffic managers during peak hours, alleviate congestion, and reduce energy consumption and environmental pollution. While this study focuses on the algorithmic design of traffic flow prediction, future work will concentrate on integrating the HGSTIN model into Intelligent Transportation Systems (ITS) to provide real-time decision support. We will further explore the hardware–software collaborative application of this model with existing traffic infrastructure to improve overall traffic management efficiency.

## Figures and Tables

**Table 1:** Description of the benchmark datasets.

Dataset	Nodes	Edges	Time Range	Time Steps
PEMS04	307	340	2018-01-01 ~2018-02-28	16,992
PEMS08	170	295	2016-07-01 ~2016-08-31	17,856

**Table 2:** Prediction performance metrics of the HGSTIN model and relevant baseline models.

Model	PEMS04			PEMS08		
	MAE	RMSE	MAPE/%	MAE	RMSE	MAPE/%
SVR	28.637	44.556	19.121	23.173	35.930	14.703
LSTM	26.865	40.963	22.458	22.239	33.617	18.838
DCRNN	22.780	36.600	14.760	18.345	28.301	11.245
STGCN	21.894	34.430	14.579	17.831	30.659	11.210
Graph WaveNet	19.420	31.700	13.330	15.075	24.044	9.563
ASTGCN	22.450	34.300	15.880	15.080	24.816	9.509
ASTGNN	18.618	31.108	12.650	14.986	27.714	9.495
HGSTIN	18.436	30.105	12.287	13.669	23.802	9.096

**Table 3:** Multi-step traffic prediction results on the PEMS04 dataset.

Model	3-Step Ahead (15 min)			6-Step Ahead (30 min)		
	MAE	RMSE	MAPE/%	MAE	RMSE	MAPE/%
DCRNN	17.500	28.483	11.633	19.078	30.986	12.346
STGCN	19.227	30.510	12.733	20.162	31.894	13.300
Graph WaveNet	17.374	28.246	11.637	17.999	29.490	12.192
ASTGCN	19.554	30.577	13.991	20.471	31.722	14.532
ASTGNN	17.643	29.288	12.176	17.960	29.908	12.318
HGSTIN	17.126	27.875	11.187	17.606	28.782	11.584

**Table 4:** Multi-step traffic prediction results on the PEMS08 dataset.

Model	3-Step Ahead (15 min)			6-Step Ahead (30 min)		
	MAE	RMSE	MAPE/%	MAE	RMSE	MAPE/%
DCRNN	16.326	25.103	10.069	16.955	26.125	10.435
STGCN	13.251	23.407	8.394	14.780	25.974	9.245
Graph WaveNet	13.000	21.524	8.415	13.752	22.463	8.739
ASTGCN	13.461	21.934	8.539	14.036	23.011	8.892
ASTGNN	13.707	22.386	8.788	14.143	23.212	9.006
HGSTIN	12.411	21.263	8.335	12.913	22.374	8.539

**Table 5:** Results of the ablation study.

Model	PeMS04			PeMS08		
	MAE	RMSE	MAPE/%	MAE	RMSE	MAPE/%
w/o Multi-feature and Multi-period Input	18.776	30.789	12.313	14.297	23.977	9.59
w/o Neighborhood Hypergraph	19.632	32.614	13.331	14.724	24.534	10.049
w/o Semantic Hypergraph	19.845	31.813	13.401	14.763	24.574	10.032
w/o Adaptive Fusion	18.523	30.142	12.324	14.087	23.712	9.680
w/o Temporal Gradient Loss	18.721	30.319	12.308	14.398	23.921	9.720
HGSTIN	18.436	30.105	12.287	13.669	23.802	9.096

## Data Availability

Data is contained within the article.
